# Impact of Silver Incorporation and Flash-Lamp-Annealing on the Photocatalytic Response of Sputtered ZnO Films

**DOI:** 10.3390/nano14181519

**Published:** 2024-09-19

**Authors:** Leo Álvarez-Fraga, Raúl Gago, David G. Calatayud, Slawomir Prucnal, Olga Sánchez

**Affiliations:** 1 Instituto de Ciencia de Materiales de Madrid, Consejo Superior de Investigaciones Científicas, Cantoblanco, 28049 Madrid, Spain; rgago@icmm.csic.es; 2 Department of Chemistry, Facultad de Ciencias, Universidad Autónoma de Madrid, 28049 Madrid, Spain; david.gcalatayud@uam.es; 3 Helmholtz-Zentrum Dresden-Rossendorf, Institute of Ion Beam Physics and Materials Research, 01328 Dresden, Germany; s.prucnal@hzdr.de

**Keywords:** co-sputtering deposition, ZnO film, silver doping, structural analysis, morphology, optical properties, flash-lamp-annealing, methyl orange, photocatalytic activity

## Abstract

Thin films of silver-doped zinc oxide (SZO) were deposited at room temperature using a DC reactive magnetron co-sputtering technique using two independent Zn and Ag targets. The crystallographic structure, chemical composition and surface morphology of SZO films with different silver concentrations were correlated with the photocatalytic (PC) properties. The crystallization of the SZO films was made using millisecond range flash-lamp-annealing (FLA) treatments. FLA induces significant structural ordering of the wurtzite structure and an in-depth redistribution of silver, resulting in the formation of silver agglomerates. The wurtzite ZnO structure is observed for silver contents below 10 at.% where Ag is partially incorporated into the oxide matrix, inducing a decrease in the optical band-gap. Regardless of the silver content, all the *as-grown* SZO films do not exhibit any significant PC activity. The best PC response is achieved for samples with a relatively low Ag content (2–5 at.%) after FLA treatment. The enhanced PC activity of SZO upon FLA can be attributed to structural ordering and the effective band-gap narrowing through the combination of silver doping and the plasmonic effect caused by the formation of Ag clusters.

## 1. Introduction

The excessive consumption of fossil fuels leading to the energy crisis and global warming has impelled researchers to look for other renewable clean energy sources. Hydrogen is a promising alternative to overcome the drawbacks of traditional fossil fuels because it has high heat conversion efficiency and zero carbon emissions. The alternative approach, known as green hydrogen, relies on the production of hydrogen via solar energy conversion through photocatalytic (PC) or photo-electrochemical (PEC) processes. Therefore, semiconductor-based photocatalysts have attracted wide attention from the scientific community to face problems dealing with hydrogen production through water hydrolysis, water waste purification and other environmental or energy-related applications. A wide variety of semiconductors (SCs), including TiO_2_ [1,2,3], ZnO, CdS and SrTiO_3_ [4,5], have been applied for photocatalytic purposes. Metal-oxide (MOX) semiconductors are mostly studied as photoanodes in P(E)C systems because they are usually environment-friendly, low-cost, stable and readily fabricated [6]. Among them, ZnO has been extensively investigated because of its band-edge position and the redox potential for photocatalytic water splitting [7]. In many cases, ZnO is more efficient as a photocatalyst than TiO_2_ because of its superior quantum efficiency, morphological versatility and low cost [8]. However, the photocatalytic hydrogen production efficiency of ZnO is significantly restricted by its intrinsic limitations, including the wide band-gap (3.37 eV) and rapid recombination of photo-generated electron-hole pairs [9]. Hence, pure ZnO may not give a desirable photocatalytic response due to reduced light absorption in the visible range so, typically, dopants are used to tune the optical band-gap of ZnO and facilitate visible light (VISL) activity [10].

Metal doping, especially with noble metals, is one of the most effective approaches to modify the optical and photodetection characteristics by introducing defect states and trap levels within the band-gap [11] reaching a remarkable improvement in the P(E)C performance under VISL irradiation. The fundamental of the plasmonic effect relies on the formation of metal nanoparticles acting as a light antenna that improves light harvesting in the VISL range. In this way, incoming light outside the band-gap of the catalyst is absorbed through the (localized) surface plasmon resonance (SPR) effect. The SPR depends strongly on the size, shape and interdistance of the nanoparticles, as well as the dielectric medium [12]. In addition to the SPR effect, the excitation of a higher number of excitons enhances the efficiency of the redox reactions [13]. Among various noble metals, silver has shown greater interest due to its surface plasmon effect within the VISL range and its natural antibacterial properties. In addition, doping with silver atoms extends the recombination lifetime of photogenerated electron and hole pairs. It also reduces the optical band-gap of the material, and consequently, the window for further applications is expanded. Doping of silver in the ZnO matrix (Ag:ZnO) can induce oxygen vacancies [14], crystal defects [15] and/or higher light scattering [16] that collectively may contribute to the enhanced photocatalytic efficiency of the photocatalytic activity of ZnO. Sputtering deposition is a widely used method for thin film growth due to its ability to cover large surface areas, making it easy to scale to an industrial level at a relatively low cost.

In this work, we used a DC reactive magnetron co-sputtering system for ZnO and Ag:ZnO thin film deposition, utilizing two separate, highly pure and independent targets of Ag and Zn. This technique allows for the control of the elemental composition of the resulting films by properly adjusting the process parameters [17]. In order to investigate the effect of silver addition on the photocatalytic properties of Ag:ZnO films, we present a comprehensive structural, morphological and optical characterization of films with different silver concentrations. Remarkably, the study is performed before and after millisecond-range flash-lamp-annealing (FLA) treatments aiming at structural ordering and doping activation.

## 2. Materials and Methods

### 2.1. Synthesis

Silver-doped zinc oxide (hereafter, referred as SZO) thin films were deposited at room temperature on (100) oriented silicon and *α*-sapphire (0001) substrates by co-sputtering from highly pure Zn (99.99%) and Ag (99.99%) 3-inch targets placed in magnetron sources. The substrate size was around 1 × 1 cm^2^. The samples with the best PC response were further evaluated with an increase in the irradiation time to 95 h and comprising larger sample areas (1.5 × 1.5 cm^2^) to increase the active surface and to be able to estimate the degradation kinetics more accurately. After pre-sputtering the targets for five minutes, the deposition process was carried out at a constant pressure of 7.4 × 10^−3^ mbar with a mixture of Ar and O_2_ gas adjusted with individual mass flow controllers (Ar/O_2_ = 4/1). The power applied to the Zn cathode (*W_Zn_*) remained constant at 100 W throughout all depositions, while the power applied to the Ag cathode (*W_Ag_*) was varied in the 0–20 W range. The reaction chamber consisted of a cylindrical vacuum chamber where two circular planar magnetrons were located in a confocal configuration with a 60° angle between each other (30° with respect the target and substrate normal for each magnetron). The distance between the center of each target and the substrate was set at 15 cm for all depositions. The base pressure was around 3.6 × 10^−6^ mbar before the deposition process. The overall deposition time for each experiment was adjusted in order to grow films with thickness in the 300–400 nm range.

After deposition, a non-contact and ultrafast (millisecond-range) thermal treatment was carried out through flash-lamp-annealing (FLA) [18]. The FLA system was based on Xe lamps with emission spectra in the wavelength range of about 250–800 nm. The flash duration was set at 23 ms under the continuous flow of O_2_ (99.999%) with an overall energy density of 40 J·cm^−2^. During the process, a peak temperature well above 1000 °C is reached in the sample according to COMSOL simulations [19]. Further details about the FLA system can be found in ref. [20].

### 2.2. Characterization

The crystalline structure of the deposited films was determined at room temperature using X-ray diffraction (XRD) (PANalytical Multi-Purpose Diffractometer model X’Pert PRO MRD) with grazing incidence geometry at an incidence angle of 0.5°. Compositional profiles were established through Rutherford backscattering spectrometry (RBS). The measurements were performed at the 5 MV HVEE Tandetron accelerator located at the Centro de Micro-Análisis de Materiales of the Universidad Autónoma de Madrid. RBS experiments were performed with ^4^He^+^ projectiles for a dose of 10 µC and energy of 1.8 MeV. The backscattered particles were detected with a silicon detector located at a scattering angle of 170°. The chemical composition of the samples has been extracted using SIMNRA 7.07 simulation software [21]. The morphology of the samples was analyzed with a field emission scanning electron microscope (FE-SEM) (Verios-460) operated at 20 kV. Sapphire substrates were used for optical transmission measurements, which were performed by using a Shimadzu SolidSpec-3700 spectrophotometer in the 200–2000 nm wavelength range. Raman experiments were performed with an Enwave EZRaman-N attached to a Leica BME microscope and equipped with a solid-state green laser (532 nm) and a CCD detector with a nominal resolution of 7 cm^−1^. The thickness of the films was determined using a Veeco Dektak 150 mechanical profilometer and confirmed via cross-sectional FE-SEM imaging.

### 2.3. PC Assessments

PC experiments were performed using a high-pressure mercury vapor lamp for UV–visible irradiation (250 W, HPL-N Philips, Amsterdam, The Netherlands). The PC performance was evaluated by immersing the samples in 5 mL of the aqueous solution of methyl orange (MO) (10^−6^ M) using a quartz beaker. Before irradiation, the samples were stirred in darkness for 60 min to ensure adsorption equilibrium. The samples are irradiated at a distance of 40 cm from the center of the lamp. During irradiation, the solution was centrifuged and sampled at regular intervals using a PerkinElmer Lambda 950 UV-Vis spectrometer. The MO solution concentrations were estimated by measuring the changes in absorbance at 465 nm. On collecting these data, two side effects must be considered, which may lead to a misinterpreted decreased value in the MO concentration: the self-degradation of the MO molecule under irradiation, as well as its incidental (partial) absorption to the surface of the samples. Here, both scenarios were discarded based on the following. On the one hand, a blank solution of MO was irradiated under the same experimental conditions, where no degradation of MO was indeed produced. On the other hand, suspensions with MO and the different samples were prepared as described before, but they were not subjected to irradiation. In such dark conditions, no changes in the MO concentration were observed, neglecting the eventual absorption of MO to the surface.

## 3. Results and Discussion

### 3.1. Composition and Growth Rate

SZO films with different Ag/Zn relative contents were deposited using the co-sputtering configuration previously mentioned. Table 1 displays the deposition conditions and thicknesses of *as-grown* SZO and after FLA films with different silver concentration. As *W_Ag_* is increased, the film thickness rises due to the higher amount of overall sputtered material. Interestingly, changes in the thickness are also observed upon FLA that will be discussed later.

**Figure 1:**
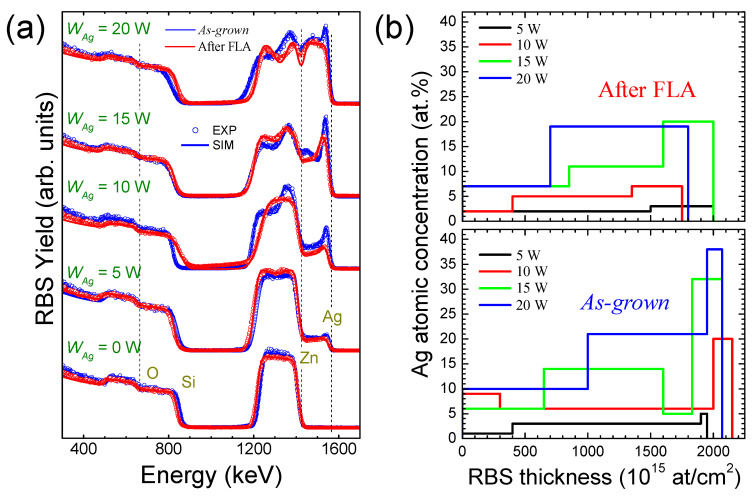
(**a**) RBS spectra of *as-grown* (blue) and after FLA (red) SZO films produced with different *W_Ag_*s. The experimental (dots) and simulated (solid lines) curves are shown for the *as-grown* samples. (**b**) Ag in-depth profiles extracted from the simulation of the RBS spectra for *as-grown* (bottom) and after FLA (top) samples.

RBS spectra and the corresponding simulation of *as-grown* and after FLA SZO films produced with different *W_Ag_* are shown in Figure 1a. The solid lines represent the simulations that reproduce the experimental data (dots), as calculated with SIMRA 7.07 software [21]. The simulated sample structure allows for extracting the overall elemental compositional profiles. The vertical dot lines indicate the projectile scattering energies from the elemental constituents in the film located at the surface where heavier elements appear at higher energies. The signal from the same element detected at lower energies would imply that the scattering is produced at a certain depth. As expected, the RBS spectra reveal an overall increase in the Ag signal with *W_Ag_*. In addition, the intensity profile anticipates the presence of inhomogeneous in-depth elemental distributions (note that there is partial overlap of the Zn and Ag signals). The spectra also indicate the Ag enrichment at the near-surface region (higher energies), which becomes more pronounced with the Ag content (*W_Ag_*). In the case of the pure ZnO film, note the shrinkage of the Zn signal after FLA, which confirms the decrease in the thickness. In the Ag-containing films, FLA also induces changes in the elemental distribution.

Note that the compositional profile is rather complex with a partial overlap of the individual signals and, hence, the simulation of the spectra is not straightforward. The simulation has been performed by considering a number of sequential layers with varying compositions in order to reproduce the experimental data. For the sake of consistency, the number of layers has been kept to the minimum possible, and most spectra can be reliably reproduced with up to three layers. The extracted Ag in-depth profiles are shown in Figure 1b. Here, both the trends in the Ag concentration and in the progressive Ag surface enrichment with *W_Ag_* are clearly evident in the *as-grown* samples. It is also remarkable that, after FLA (top panel), the Ag distribution becomes more homogeneous.

The quantitative results from the RBS simulation are displayed in Table 2. Here, the atomic incorporation rate has been extracted from the atomic areal density and the deposition time. We can clearly see the increase in the incorporation rate with *W_Ag_* as more material is sputtered. Note that the changes in the thickness observed after FLA (Table 1) also reflect on the incorporation rate obtained via RBS. The average silver content within the film increases with the *W_Ag_* with values ranging from 3 to 17 at.% for *as-grown* samples. In this case, there is a slight decrease in the Ag content after FLA. For pure ZnO, a nearly stoichiometric film is obtained. In addition, oxygen uptake seems to be related to the Ag content. This trend could be explained by the formation of a more porous structure as silver is increased. Indeed, this can be supported by the strong decrease in the film density for *W_Ag_* > 15 W. The density in the films was determined by the atomic areal density extracted from the RBS measurements (expressed in 10^15^ at/cm^2^) and the film thickness. For reference, the density value of bulk ZnO is 5.6 g/cm^3^ [22]. Note also the decrease (increase) in the density for low (high) Ag contents after FLA that will be discussed later.

### 3.2. Structural Analysis

**Figure 2:**
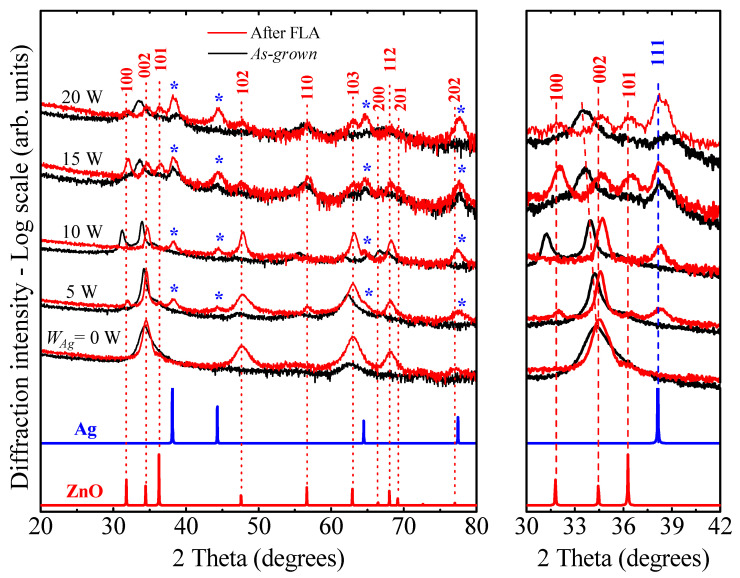
XRD pattern (with a logarithmic scale to evidence possible weak peaks) of SZO films varying the Ag concentration, and several references as ZnO and Ag (marked with *). At the right side, a magnified view of the main diffraction maximum around the 2*θ* value range between 30° and 42°.

Figure 2 shows the XRD patterns for the both SZO thin films, *as-grown* (blue lines) and after FLA (red lines) with different Ag concentrations. The reflections from the reference spectra of wurtzite ZnO (JCPDS card no. 36-1451) and Ag (JCPDS card no. 04-0783) are also included for discussion. The undoped ZnO film has a hexagonal wurtzite structure with a dominant (002) reflection. It is also possible to observe the presence of a (103) peak. Note that the *y*-axis of the graph is presented with a logarithmic scale to evidence possible weak peaks.

The film structure changes slightly with low Ag doping (*W_Ag_* ≤ 10 W). In this case, other weak diffraction peaks related to the ZnO structure appear, suggesting the formation of more randomly oriented grains through Ag incorporation. In addition, there is a narrowing of the (002) peak as extracted from the full-width at the half maximum (FWHM), indicating an improved crystallinity for low doping levels (see details in Table 3). Within this Ag range (*W_Ag_* ≤ 10 W), no signal related to metallic silver or silver oxides was detected, indicating that silver does not segregate and could be incorporated into the ZnO matrix. This assumption is confirmed by the progressive shift of the (002) peak to lower Bragg angles with the silver content, suggesting the substitutional incorporation of Ag since its ionic radius (144 pm) is larger than that of Zn^2+^ (135 pm). Apart from the shift, there is a progressive peak broadening that indicates that the wurtzite structure becomes distorted as more Ag is incorporated in the oxide matrix. The disruption of the hexagonal wurtzite-ZnO structure occurs for *W_Ag_* > 10 W, as the signal from the diffraction peaks related to this phase becomes broader and weaker, until it completely vanishes. Instead, new additional reflections ascribed to the cubic phase of metallic silver appear (indicated with blue asterisks), indicating the agglomeration or clustering of silver atoms.

After FLA, significant structural changes occur. First, for samples with no or very low Ag contents (*W_Ag_* ≤ 5 W) there is a narrowing of the (002) reflection from ZnO, indicating significant structural ordering. The appearance of additional ZnO reflections, with respect to the *as-grown* samples, indicate that such ordering results in the formation of randomly oriented grains. The appearance of almost all ZnO peaks is also observed in all the films, indicating a general transformation towards a more stable ZnO-based (random) structure, which is noteworthy. This trend is accompanied by a redistribution of the Ag atoms after FLA in line with the RBS data, where less Ag is retained inside the ZnO grains as extracted from the shift of the (002) peaks to higher scattering angles. Accordingly, the intensity of Ag peaks increases with *W_Ag_*, and a randomly oriented wurtzite ZnO-based structure is formed. This situation reveals the presence of metallic Ag clusters as a secondary crystalline phase within the oxide matrix. The shift of the (002) peak to higher Bragg angles and its narrowing indicates a possible contraction in the interplanar distance, which could be an indication of higher compactness of the crystalline structure. The difference between the atomic radius of Ag^+^ and Zn^2+^ limits the solubility of Ag in the ZnO lattice site [23]. Hence, Ag accommodation within the wurtzite phase after FLA seems to depend on the overall Ag content, and this effect is more evident for the sample grown with *W_Ag_* = 10 W. The estimated values of the lattice constants (*a* and *c*) obtained from the (002) and (103) planes, as well as the corresponding *c/a* ratio, crystallite sizes and in-plane stress (*σ*) of SZO films with low Ag contents, are presented in Table 3.

The crystallite sizes of the wurtzite phase were calculated using Scherrer’s equation [24], based on the broadening of the (002) peak. The grain size of the *as-grown* films depends on the Ag content. The crystallites grow bigger with the increase in the Ag content and reach a maximum value of 25.50 nm. After FLA, the crystallite sizes were further increased to 14.53 nm for the undoped ZnO film and to 29.72 nm for *W_Ag_* = 5 W. Then, it decreased to 19.67 nm for *W_Ag_* = 10 W. Similar trends have been found after FLA for other doping elements in ZnO, such as in the case of aluminum [25]. The lattice parameters for the ZnO wurtzite structure were calculated from the position of the XRD reflections. These values are comparable to the lattice constants of ZnO bulk (*C*_0_) at 5.206 Å [22]. The evolution of lattice parameters for the films with low Ag contents are also shown in Table 3. All deposited SZO films showed *c* parameter values slightly higher than those of unstressed ZnO, indicating that the unit cells are more elongated along the *c*-axis. This effect is higher as the Ag content increases. On the contrary, after FLA, all the SZO films show a decrease in the *c* parameter, with values slightly lower than in the (relaxed) wurtzite ZnO structure. Note that the decrease is higher as the Ag content increases.

It is well established that the lattice distortions due to the defects (vacancies, interstitials, substitutions, local structure transformations, etc.) may cause strain in the films. Depending on the type of strain in the crystal, i.e., tensile or compressive strain, the peak position shifts towards higher or lower angles, respectively [26]. For the hexagonal crystal structure, the in-plane stress (*σ*) of the films can be calculated using the biaxial strain model [27]: *σ* = −450·(*C*_0_ − *C*)/*C*_0_, where *C*_0_ (5.206 Å) is the unstrained lattice constant for the powder reference [22], and *C* is the lattice constant that was obtained experimentally. The negative sign in biaxial stress (*σ*) values indicated that all the *as-grown* SZO films were in a state of compressive stress. Depending on the size of the foreign impurity atom and the size of the host atom, the compressive stress may increase or decrease [28]. It is seen from Table 3 that the stress in as-*as-grown* SZO films showed a tendency to increase with the rise in the Ag content. After FLA, the internal stress turns into a tensile state. The change in stress upon FLA can be attributed to the promotion of interstitial oxygen as suggested by Gupta et al. [29]. Moreover, different thermal expansion coefficients (*α*) between Si (*α_Si_* ~ 3 × 10^−6^ K^−1^) and ZnO that have different expansions along different axes (*α_ZnO-a_* ~ 6.5 × 10^−6^ K^−1^ along the *a*-axis and *α_ZnO-a_* ~ 3.7 × 10^−6^ K^−1^ along the *c*-axis) may play a role. During the FLA process, the lattice parameter of ZnO along the *a*-axis expands more than that along the *c*-axis, and for the *a*-axis, the expansion is much bigger than for Si. The heating rate for ms-range annealing is about 5 × 10^4^ K/s, and the cooling rate is about 20 K/s. Due to this fast cooling rate, the lattice extension of the unit cell is maintained (bigger along the *a*-axis than for the *c*-axis) causing in-plane tensile strain. The same phenomenon is observed for Ge thin films (*α_Ge_* ~ 6 × 10^−6^ K^−1^) grown on Si [30]. In the *as-grown* state, the Ge layer is compressive strained, while after fast cooling, the significant difference in *α* between Ge and Si frustrates the shrinkage of Ge and the grown layer becomes tensile strained. The biaxial tensile stress in the films is found to be slightly increased by the silver incorporation (see Table 3).

### 3.3. Morphological Study

**Figure 3:**
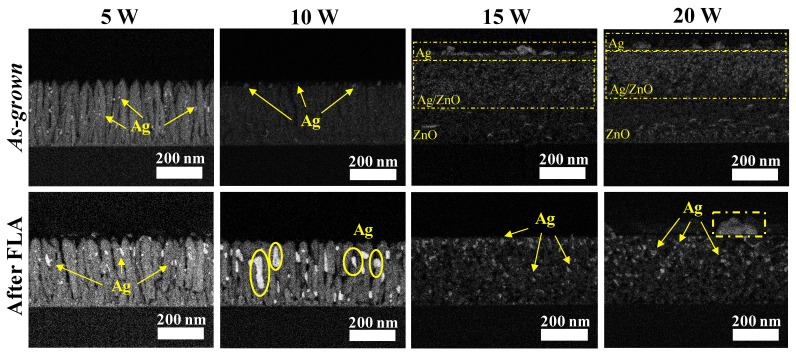
Cross-sectional BSE images of SZO films with different Ag contents.

It is well known that ZnO films deposited normal to the substrate exhibit a columnar microstructure oriented perpendicular to the silicon substrate [31]. The back-scattered electron (BSE) signal from the cross-section view of the *as-grown* SZO and after FLA films with different silver contents are displayed in Figure 3. In this case, the image contrast provides additional compositional information, since a brighter signal can be related to the presence of heavier elements. Initially, for very low silver contents (*W_Ag_* = 5 W), the significant presence of bright spots (related to small Ag aggregates) started to appear taped throughout the columnar structures. Increasing the Ag content in the films grown at *W_Ag_* = 10 W leads to a more pronounced segregation of silver particles towards the ZnO surface, in agreement with the RBS data. Note also the preferential formation of well-defined silver-rich regions on the edges of the ZnO columns. When the silver content is higher (*W_Ag_* ≥ 15 W), the topography and microstructure of the films are quite different. First, no columnar structure is observed, and it is possible to appreciate three distinct layers with a presumably different composition, as evidenced by the change in the image contrast. A proposed structure, in accordance with the RBS results, consists of a superficial layer of mainly silver (brighter), an intermediate layer of Ag and ZnO and a deeper layer richer in ZnO.

After FLA, SZO films with low silver doping evidence a severe atomic diffusion, promoting the agglomeration of silver and formation of larger particles. In particular, for *W_Ag_* = 10 W, a significant migration of silver towards the grain interfaces (between columns) is observed, accompanied by a substantial increase in the size of the Ag particles. Note that this process seems to leave the formation of voids in the films, resulting in open pores that could be relevant for the PC activity. The formation of these voids can also be related to the thickness increase after FLA. The growth and agglomeration of silver particles after FLA can be attributed to the Ostwald ripening mechanism [32] where larger particles grow at the expense of smaller ones due to differences in their surface energy. The high temperature influences Ostwald ripening due to its effect on interfacial energy, coefficients of the growth rate and solubility [33]. When the Ag content is higher (*W_Ag_* ≥ 15 W), FLA promotes extensive diffusion and the reorganization of atoms (a substantial decrease in thickness is also observed), leading to the formation of a single mixed layer.

**Figure 4:**
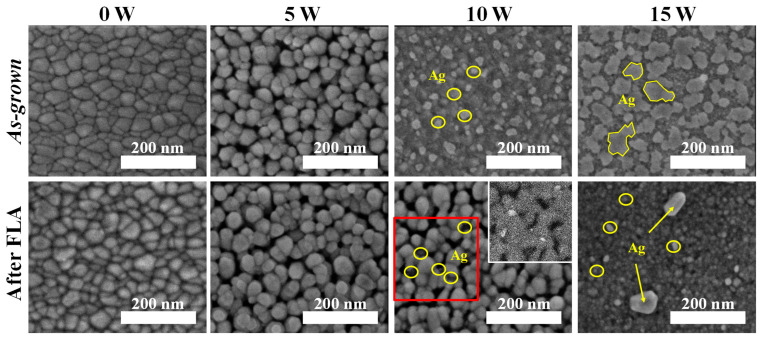
Top-view SEM images of *as-grown* and after FLA SZO films with different Ag doping levels. For comparison, a BSE image of the region marked in red is provided as the inset.

The top-view SEM images of SZO films with different silver contents are shown in Figure 4. It is clear that the surface morphology changes considerably with the Ag incorporation. The undoped ZnO film presents a dense granular structure composed of relatively large grains (40 ± 5 nm) that can be ascribed to the columnar growth. For very low Ag doping (*W_Ag_* = 5 W), a morphological transition to a more porous structure is observed. The absence of defined silver particles on the surface suggests limited Ag agglomeration and, hence, a uniform distribution of Ag within the ZnO matrix. The subsequent increase in the doping for *W_Ag_* = 10 W results in pronounced silver accumulation on the surface, overshadowing the ZnO structure. A further increase in the doping level (*W_Ag_* = 15 W) leads to the formation of silver patches on the surface. After FLA for low Ag dopings (*W_Ag_* ≤ 10 W), it is evident that the empty space between the grains increases, which, as indicated above, can be related to a higher porosity. This change is due to the silver segregation and redistribution along the columns, similarly to the behavior reported by R. Francq et al. [34]. In particular, it is observed that, in the film with *W_Ag_* = 10 W, the oxide grains are revealed after FLA as a result of silver migration from the surface (as previously noted in the cross-sectional images). An inset corresponding to a high-contrast BSE image of the region marked in red is provided for distinguishing both phases (Ag and ZnO). Regions of silver are detected, but no longer on the surface and rather embedded within the columns. Increasing the Ag content (*W_Ag_* = 15 W) results in a larger particle size accompanied by a dense array of Ag nanoparticles on the sample surface.

### 3.4. Optical Properties

**Figure 5:**
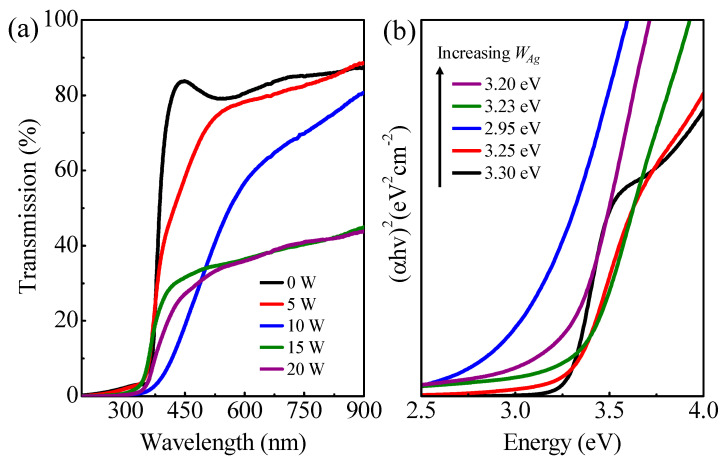
(**a**) Optical transmittance spectra; (**b**) the Tauc plot of (*αhv*)^2^ vs. energy *hv* to determine the band-gap energy.

Figure 5a shows the optical transmittance spectra of the full set of *as-grown* SZO films on sapphire. Undoped ZnO and low-Ag-content films (*W_Ag_* ≤ 10 W) show good optical transmittance values of 75–85% over the whole range of visible wavelengths. It is obvious that, with a rising Ag doping content, the transmittance gradually decreases until it reaches a fixed value below 40% (*W_Ag_* ≥ 15 W), revealing the more metallic character of the films. In order to calculate the band-gap energy (*E_g_*), one of the methods mostly used is based on the Tauc model [35]. In this case, *E_g_* is extracted from the following expression: *αhv~(hv-E_g_)^n^*, where *α* is the optical absorption coefficient, *h* is Planck’s constant, *v* is the frequency of radiation and the exponent *n* depends on the nature of optical transition. In the present case, *n* = 2, since the optical absorption edge is commonly described by an allowed direct transition. The *E_g_* of the SZO film can be estimated by plotting (*αhv*)^2^ vs. *hν* and extrapolating the best linear fit with the abscissa axis. Figure 5b shows the corresponding Tauc plots with the variation in *E_g_* as a function of the Ag contents. The calculated *E_g_*s are listed as the inset in Figure 5b. Compared to the undoped ZnO film with an *E_g_* of ~3.30 eV, the optical absorption edge exhibits a red-shift with the rise in Ag doping content up to *W_Ag_* = 10 W. The narrowing of *E_g_* could be due to the incorporation of Ag^+^ into the ZnO matrix, altering the band structure, as supported by the XRD data. Ag atoms play an important role as an acceptor to decrease the *E_g_* of ZnO [36]. However, *E_g_* increases for *W_Ag_* ≥ 15 W as a result of the disruption of the wurtzite structure and the diffusion of Ag particles towards the ZnO surface, as observed via XRD and SEM.

**Figure 6:**
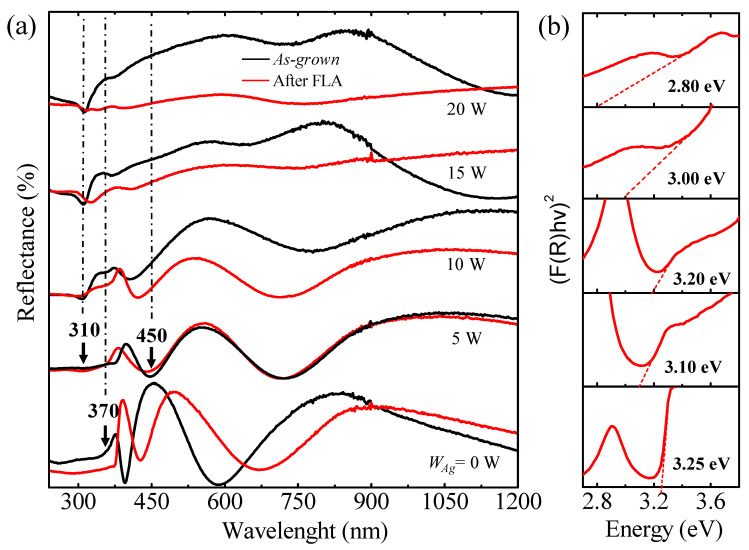
(**a**) Direct reflectance spectra of SZO films with different Ag contents; (**b**) Kubelka–Munk plot of reflectance spectra to determine the band-gap energy.

Since FLA has been applied to films grown on silicon substrates, reflectivity has been used to study the impact of the thermal treatment in the optical properties. The reflectance spectra of *as-grown* and after FLA SZO films are shown in Figure 6a. The broad strong intense absorption edge position around 370 nm, characteristic of ZnO direct band-gap transition, is related to the charge-transfer mechanisms from the valence band state to the conduction band states of the ZnO interface. The presence of interference fringes is also indicative of the transparency of the films, which is maintained for *W_Ag_* ≤ 10 W. Silver incorporation reveals a distinctive peak at 310 nm due to interband transition in Ag nanoparticles, which becomes more pronounced upon the Ag increase [37]. A noticeable broad band at 450 nm is associated with the SPR peaking of metallic Ag particles [38]. The blueshift of this band can often indicate structural and electronic modifications. In order to calculate the *E_g_* from the reflectance spectra, we have considered the Kubelka–Munk function (*F_KM_*) given by the following expression [39]: *F_KM_*(*R*) = (1 − *R*)^2^/2*R*, where *R* is the reflection coefficient obtained from the spectra. The linear part of the curve was extrapolated to *F_KM_* = 0 to obtain the *E_g_* of the films. It is worth mentioning that the *E_g_* of the *as-grown* films, calculated using this method, match those calculated using the Tauc method (they are not included here). Figure 6b shows the *E_g_* plot calculated from the corresponding reflectance spectra using the Kubelka–Munk method in the case of FLA samples. The resulting plots show that the absorption edge after FLA generally shifts towards lower energies, which may be attributed to the Ag redistribution and/or change in the stress state from compressive to tensile, as suggested by the XRD results. In any case, the decrease in *E_g_* after the thermal treatment is in agreement with the report of Tran et al. [40]. However, the film with *W_Ag_* = 10 W displays an increase in the *E_g_* from 2.95 to 3.20 eV after FLA.

### 3.5. Photocatalytic Activity

**Figure 7:**
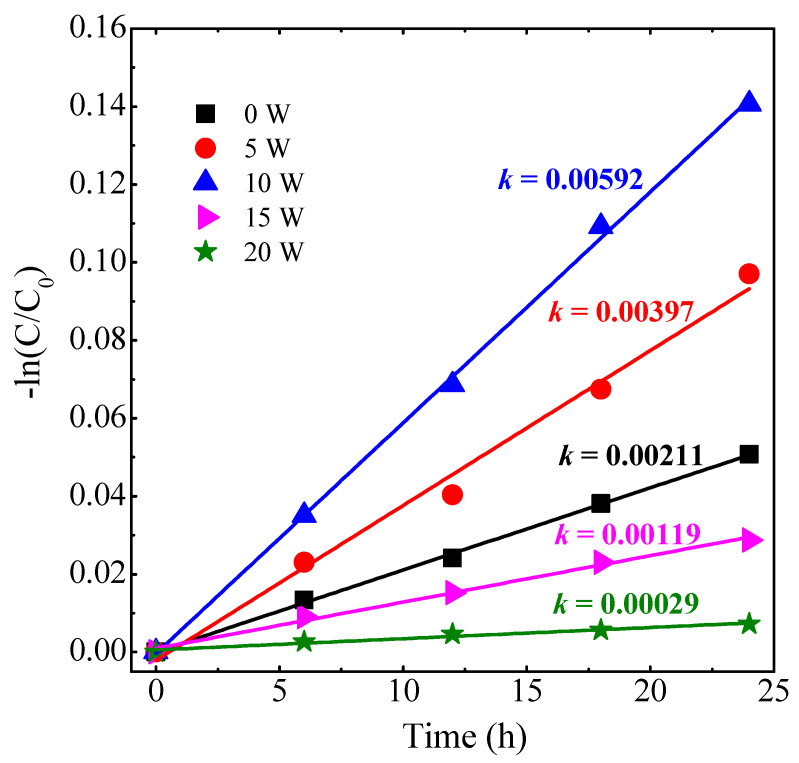
Kinetic plot of photocatalytic degradation of MO using SZO thin films under irradiation.

The PC activity of all SZO thin films was evaluated based on MO degradation under UV–vis irradiation with a light bulb. It is important to mention that, regardless of the Ag content, all the *as-grown* SZO films (i.e., untreated with FLA) do not exhibit any significant PC activity. This suggests that FLA is crucial for activating the PC activity in the films, which can be attributed to the structural and morphological changes described above. Figure 7 shows the reaction kinetics for different SZO films. The slope of the curves (*k*) can be related to the first-order rate constant for the degradation process, for which values are also indicated in the figure. Here, the ZnO films become photoactive after FLA, which can be related to the ordering increase in the wurtzite structure. Noteworthy, the samples with low Ag contents (*W_Ag_* ≤ 10 W) show the major increase in the photoactivity, with a maximum yield for *W_Ag_* = 10 W. For higher Ag contents (*W_Ag_* ≥ 15 W), the films are photoactive but the yield decreases with respect to the other SZO samples and is even lower when compared with the undoped ZnO case.

**Figure 8:**
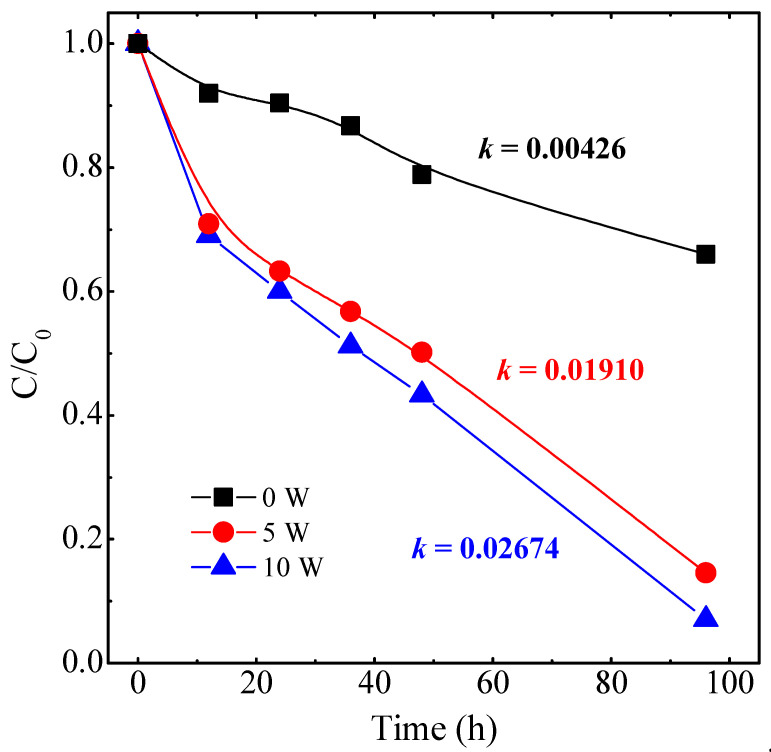
Concentration decay curves of MO for selected samples with larger surface areas.

Taking into consideration the preliminary results shown in Figure 7, the best samples were further evaluated with an increase in the irradiation time to 95 h and comprising larger sample areas to increase the contact effective surface and to be able to estimate the degradation kinetics more accurately. The PC conditions were kept identical as in the first experiment. Figure 8 shows the irradiation time dependence of the MO photodegradation process. After 95 h of irradiation, the best photocatalytic result was again attained in the film with *W_Ag_* = 10 W, reaching up to~93% of the MO concentration reduction.

Based on the structural and optical properties reported here, the superior photoactivity in SZO samples after FLA can be ascribed to the combination of structural ordering of the wurtzite matrix, enhanced optical absorption by the band-gap narrowing and agglomeration (plasmonic effect) of Ag, together with significant porosity. Obviously, the out-diffusion of Ag and formation of the metal-rich surface layer at high doping levels seems to be detrimental for the PC activity, which may be related to a masking effect of Ag on ZnO-based grains preventing or blocking the presence of PC active sites on the sample surface. On the other hand, it has been observed that the wettability of the samples is dependent on the Ag content (i.e., the wettability can be tuned with the Ag content) [17]. In this case, the films change from hydrophobic (*CA* = 124°) to hydrophilic (*CA* = 85°) upon FLA, which can be relevant to explain the PC activation. This improved wettability allows for faster and more effective contact between the surface of the material and the dye to be degraded, resulting in early photoactivity. Thus, the process is not delayed by the time needed for more hydrophobic samples to become wet. Moreover, the surface roughness values (4 ± 2 nm rms) determined via optical profilometry show only small deviations between all samples. So, this parameter does not seem to be an important factor to explain the different PC behavior with the Ag content.

**Figure 9:**
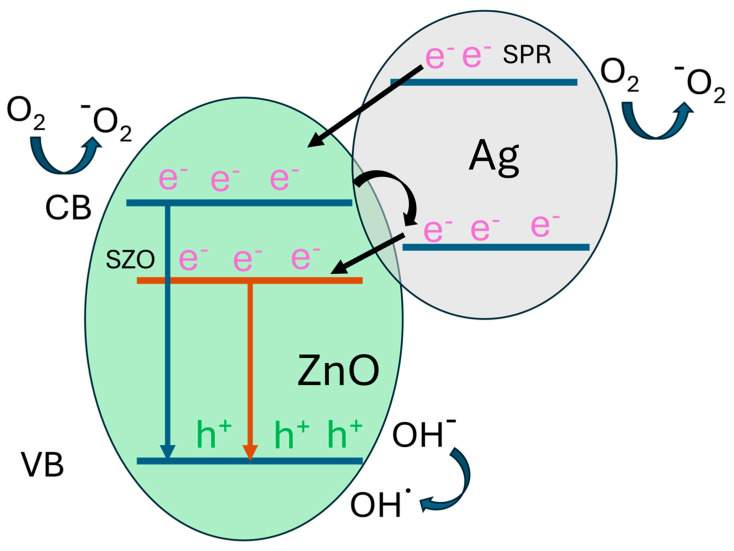
Proposed mechanism behind the photocatalytic degradation activity of hybrid Ag/ZnO nanostructures.

Therefore, it is confirmed that the good PC properties are based on a compromise between the ZnO structure and the percentage of silver, reaching the maximum PC when the balance between the non-defective structure and the defects generated by the silver doping are in the right ratio. The PC process depends on the light absorption efficiency, crystallinity and surface area [41,42], and by optimizing these factors, the efficiency of semiconductor photocatalysts can be significantly improved, leading to better performance in applications, such as water splitting and pollutant degradation, among others. Also, a good balance between these factors is desirable to improve the photoactivity. If we analyze these parameters in detail, we can say the following: (i) light absorption efficiency determines how well the semiconductor can absorb photons to generate electron–hole pairs. Materials with a broad absorption spectrum, especially in the visible range, are more efficient. Enhancing light absorption can be achieved by doping or creating heterojunctions and, in our case, silver incorporation does so. (ii) High crystallinity typically means fewer defects, which can act as recombination centers for electron–hole pairs. Better crystallinity improves charge carrier mobility, leading to more efficient PC reactions. For that reason, the higher content of silver, the more defects in the structure, and therefore, this causes a reduction in photoactivity. (iii) A larger surface area provides more active sites for the photocatalytic reactions. Nanostructured materials, such as nanoparticles or nanorods, often exhibit higher surface areas, enhancing their photocatalytic performance. In our case, due to the morphological characteristics of our surfaces, this is improved with respect to the bulk material. The mechanism behind the PC degradation activity of SZO samples can also be sustained on the formation of an heterostructure, as explained in the following. In this way, there are three possible electron-transfer mechanisms [43] at the metal–semiconductor interface as depicted in Figure 9. Electron transfer through these mechanisms would mitigate the recombination of charge carriers and, hence, they become available to participate in the PC process.

In order to confirm the proposed PC mechanisms, the impact of FLA on the defect structure of the wurtzite phase has been addressed via Raman spectroscopy. In this case, we focus on low Ag contents where we have obtained the higher PC, and obviously, the wurtzite structure is maintained upon doping. It is well known that wurtzite ZnO has eight sets of characteristic optical phonon modes at the center of the Brillouin zone (*Г* point), given in equation [44]: *Г* = *1A*_1_ *+* 2*B*_1_ *+* 1*E*_1_ *+* 2*E*_2_, where *A*_1_ and *E*_1_ modes are polar and split into the transverse optical mode (TO) and a longitudinal optical mode (LO). *E*_2_ mode consists of *E*_2_ (low) associated with the vibration of the oxygen atom and *E*_2_ (high) related to a heavy Zn sublattice. In addition, the *B*_1_ modes are generally not Raman active.

**Figure 10:**
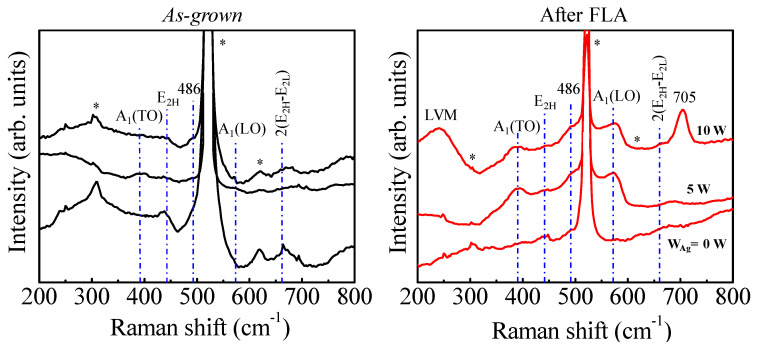
Raman spectra of SZO films *as-grown* and after FLA, varying the Ag content. The Raman vibration modes are included in the figure. Peaks marked with an asterisk are attributed to scattering from the silicon substrate.

Figure 10 shows the Raman spectra of *as-grown* (left) and FLA (right) samples. The undoped ZnO film presents a characteristic phonon mode situated at 438 cm^−1^ assigned to the vibrational mode *E*_2*H*_ related to the crystalline nature, phase orientation and strain state present in the ZnO matrix [45]. The intensity of this peak decreases and broadens with Ag incorporation, which indicates a more disordered wurtzite structure in line with the XRD results. Additionally, the Raman peak at 486 cm^−1^ exclusively appeared for Ag-doped ZnO, due to the interfacial surface phonon mode [38].

After FLA, the *A*_1_*(TO)* and *A*_1_*(LO)* polar branches appeared at about 380 and 570 cm^−1^, respectively, for Ag-doped ZnO. The *A*_1_*(LO)* mode is commonly related to the presence of defects, assigned to oxygen vacancies and zinc interstitials in ZnO. These defects seem to be promoted with the Ag content. In addition, it is observed that in the film with *W_Ag_* = 10 W, a complementary peak appears: a broad Raman peak at 241 cm^−1^ attributed to the local vibrational mode (LVM) [46]. This feature is accompanied by an intense peak at 705 cm^−1^, which may be related to the local vibrational mode of the defect/impurity in Ag-doped ZnO [47]. Accordingly, as discussed above, this situation would support the good PC activity of the FLA film with *W_Ag_* = 10 W towards MO degradation under UV–vis irradiation. Therefore, the good PC properties rely on a compromise between the quality of the ZnO structure and the percentage of silver. Under this framework, the maximum photoactivity would be reached as a result of the balance between the non-defective wurtzite structure and the defects generated by the silver doping.

## 4. Conclusions

We used a DC magnetron co-sputtering system for tuning the Ag content in ZnO films from two highly pure and independent Ag and Zn targets. In order to further investigate the effect of Ag addition on the PC properties of ZnO films, we have carried out a structural, morphological and optical characterization of ZnO films with different Ag concentrations before and after millisecond-range FLA treatment. The PC activity of the all SZO thin films was evaluated based on MO degradation under UV–vis irradiation with a light bulb that mimics the solar spectrum. It is important to mention that, regardless of the silver content, all the *as-grown* films (i.e., untreated with FLA) do not exhibit any significant PC activity. This suggests that FLA is crucial for activating the PC response of the films, which can be attributed to the induced structural and morphological changes. The samples grown with *W_Ag_* ≤ 10 W and after FLA show the highest PC activity, where the yield increases with the Ag incorporation within this range. For a higher silver content (*W_Ag_* ≥ 15 W), there is a decrease in the PC activity, even lower than that of FLA undoped ZnO. Based on the structural and optical properties, the superior photoactivity in the samples after FLA can be ascribed to the combination of structural ordering of the ZnO matrix and enhanced optical properties mediated by Ag doping and agglomeration (plasmonic effect), together with a significant porosity. Obviously, the out-diffusion of Ag and formation of a metal-rich surface layer at high doping levels seems to be detrimental for the PC activity, which may be related to a masking effect of silver over ZnO grains preventing or blocking the presence of PC active sites on the surface.

## Figures and Tables

**Table 1:** Deposition conditions and thickness for the SZO thin films before and after FLA.

*W_Zn_*	*W_Ag_*	Time	Thickness (nm)	
(W)	(W)	(min)	*As-grown*	After FLA
100	0	62	230 ± 23	270 ± 27
100	5	47	300 ± 30	310 ± 31
100	10	42	280 ± 28	310 ± 31
100	15	38	400 ± 40	320 ± 32
100	20	35	460 ± 46	320 ± 32

**Table 2:** Fitting results of the RBS spectra for SZO thin films as a function of *W_Ag_*.

*W_Ag_* (W)	*As-grown*				After FLA			
	Inc. Rate (at.nm^2^s)	Ag (at.%)	O/Zn	Density (g/cm^3^)	Inc. Rate (at.nm^2^s)	Ag (at.%)	O/Zn	Density (g/cm^3^)
0	4.8 ± 0.1	-	1.0 ± 0.1	5.1 ± 0.5	5.2 ± 0.1	-	1.1 ± 0.2	4.7 ± 0.5
5	6.9 ± 0.2	3 ± 1	1.2 ± 0.2	4.4 ± 0.5	7.1 ± 0.2	2 ± 1	1.2 ± 0.2	4.3 ± 0.5
10	8.5 ± 0.2	7 ± 1	1.6 ± 0.2	5.1 ± 0.5	7.6 ± 0.2	5 ± 1	1.0 ± 0.1	4.5 ± 0.5
15	9.1 ± 0.2	13 ± 1	1.7 ± 0.3	3.8 ± 0.5	8.8 ± 0.2	11 ± 1	1.5 ± 0.2	4.5 ± 0.5
20	9.9 ± 0.2	17 ± 1	1.8 ± 0.3	3.5 ± 0.5	8.6 ± 0.2	14 ± 1	1.6 ± 0.2	4.2 ± 0.5

**Table 3:** The average crystallite size, lattice parameters and in-plane stress (*σ*) of SZO thin films doped with low Ag concentration *as-grown* and after FLA.

*W_Ag_* (W)	Conditions	2θ_pos_ (002)	*D* (nm)	Lattice Parameters		*c*/*a* Ratio	*σ* (GPa)
				*a* = *b* (Å)	*c* (Å)		
-	ZnO bulk	34.422	-	3.250	5.205	1.602	-
0	*As-grown*	34.340	9.88	3.274	5.223	1.595	−1.47
5		34.229	23.14	3.300	5.239	1.587	−2.85
10		33.958	25.50	3.330	5.276	1.584	−6.05
0	After FLA	34.529	14.53	3.251	5.195	1.598	+0.95
5		34.578	29.72	3.265	5.188	1.589	+1.55
10		34.698	19.67	3.266	5.170	1.583	+3.11

## Data Availability

The data presented in this study are available in this article.
